# Inotrope Use and Intensive Care Unit Mortality in Patients With Cardiogenic Shock: An Analysis of a Large Electronic Intensive Care Unit Database

**DOI:** 10.3389/fcvm.2021.696138

**Published:** 2021-09-21

**Authors:** Fei Gao, Yun Zhang

**Affiliations:** Department of Emergency Medicine, Wuxi People's Hospital Affiliated to Nanjing Medical University, Wuxi, China

**Keywords:** inotrope, cardiogenic shock, intensive care unit, mortality, outcome

## Abstract

**Purpose:** To determine whether inotrope administration is associated with increased all-cause mortality in cardiogenic shock (CS) patients and to identify inotropes superior for improving mortality.

**Methods:** This retrospective cohort study analyzed data retrieved from the Philips Electronic ICU (eICU) database, a clinical database of 200,859 patients from over 208 hospitals located throughout the United States. The database was searched for patients admitted with CS to the intensive care unit (ICU) between 2014 and 2015. We evaluated 34,381 CS patients. They were classified into the inotrope and non-inotrope groups based on whether inotropes were administered during hospitalization. The primary endpoint was all-cause hospital mortality.

**Findings:** In total, 15,021 (43.69%) patients received inotropes during hospitalization. The in-hospital mortality rate was significantly higher in the inotrope group than in the non-inotrope group (2,999 [24.03%] vs. 1,547 [12.40%], adjusted hazard ratio: 2.24; 95% confidence interval [CI]: 2.09–2.39; *p* < 0.0001). After propensity score matching according to the cardiac index, 359 patients were included in each group. The risk of ICU (OR 5.65, 95% CI, 3.17–10.08, *p* < 0.001) and hospital (OR 2.63, 95% CI: 1.75–3.95, *p* < 0.001) mortality in the inotrope group was significantly higher. In the inotrope group, the administration of norepinephrine ≤0.1 μg/kg/min and dopamine ≤15 μg/kg/min did not increase the risk of hospital mortality, and milrinone administration was associated with a lower mortality risk (odds ratio: 0.559, 95% CI: 0.430–0.727, *p* < 0.001). Meanwhile, the administration of >0.1 μg/kg/min dobutamine, epinephrine, and norepinephrine and dopamine >15 μg/kg/min was associated with a higher risk of hospital mortality.

**Conclusions:** Inotropes should be used cautiously because they may be associated with a higher risk of mortality in CS patients. Low-dose norepinephrine and milrinone may associated with lower risk of hospital mortality in these patients, and supportive therapies should be considered when high-dose inotropes are administered.

## Introduction

Cardiogenic shock (CS) is a life-threatening condition caused by a primary cardiac disorder. CS is characterized by persistent hypotension that is unresponsive to volume replacement and is accompanied by clinical features of end-organ hypoperfusion. It requires pharmacological intervention or mechanical support (1). Positive inotropic drugs are typically used to stabilize patients with CS in the intensive care unit (ICU) as a bridge to heart replacement therapy or to decision. However, clinical studies have failed to demonstrate the benefits of these agents. Further, inotropes have been reported to be associated with an increased risk of adverse outcomes (2–4), and insufficient data are available on clinical outcomes to guide the initial selection of inotropic drugs in patients with CS.

The Sepsis Occurrence in Acutely Ill Patients II Trial showed that dopamine is associated with a higher rate of arrhythmias in CS patients and the overall population, as well as a higher risk of mortality in the CS subgroup (5). Although this was the largest study of its kind, clinical and methodological issues in the study have raised concerns about its external validity and the applicability of its findings in CS patients. Another single-center retrospective study based on cardiac ICU data showed that increased use of peak vasopressors and inotropes was strongly associated with hospital mortality. Further, the use of norepinephrine was associated with lower mortality among patients requiring higher vasopressor doses (6). However, this study only observed the prevalence of vasoactive drug use in the cardiac ICU population without analyzing the association between inotrope use and outcomes in CS patients.

Current recommendations are based mainly on results of meta-analyses and expert opinions. The French, Scandinavian, and German recommendations are very similar and unanimously recommend norepinephrine and dobutamine as first-line agents (7, 8), and the American Heart Association also continues to advocate dopamine use in CS (9). However, there is insufficient evidence to prove that any single inotrope is superior to another in terms of improving mortality (10, 11).

As such, this study aimed to (1) examine the association between inotrope use and hospital mortality or other clinically important endpoints in CS patients admitted to the ICU, (2) identify risk factors of hospital mortality in CS patients, and (3) identify inotropes that are superior in improving mortality outcomes in CS patients.

## Materials and Methods

### Study Design and Database

This retrospective cohort study analyzed data retrieved from the Philips Electronic ICU (eICU) database, a clinical database of 200,859 patients from over 208 hospitals located throughout the United States. All patients were admitted to ICUs between 2014 and 2015. The database is publicly accessible, and an online training course is required before accessing it. YZ obtained certification from the Massachusetts Institute of Technology (certification number: 36026306) after successfully completing the CITI program. Thereafter, YZ received approval to access and use the eICU database. The need for informed consent was waived as this was an independent research analysis of already available data. All data were anonymized by an eICU programmer prior to the commencement of the analysis.

### Study Participants

We evaluated 34,381 patients diagnosed with CS between 2014 and 2015. The inclusion criterion was age ≥ 18 years. Only data from first ICU admissions were used for patients with subsequent readmissions. CS patients were identified according to the International Classification of Diseases, 9th Edition codes (ICD-9) 758.51 and R57.0 and divided into the inotrope and non-inotrope groups based on whether or not inotropic agents were used during hospitalization.

### Definition of Variables

The primary endpoint was all-cause hospital mortality, and the secondary endpoints were all-cause ICU mortality, the incidence of severe acute renal failure (ARF), ICU length of stay (LOS), duration of ventilation, and hospital LOS. Data on the type and quantity of inotrope administered for the duration of hospitalization were obtained from the eICU database.

The administered inotropes included dobutamine, dopamine, epinephrine, norepinephrine, and milrinone. All inotropes were administered via continuous infusion. Low-dose (LD), middle-dose (MD), and high-dose (HD) dopamine were defined as peak doses of ≤5, 5–15, and >15 μg/kg/min, respectively. LD and HD norepinephrine were defined as peak doses of ≤0.1 and >0.1 μg/kg/min, respectively. LD and HD epinephrine were defined as peak doses of ≤ 0.1 and >0.1 μg/kg/min, respectively.

Controlled variables were age, sex, body mass index (BMI), disease severity scores (Acute Physiology and Chronic Health Evaluation IV [APACHE-IV] score and simplified Acute Physiology Score II [APS-II]), and the presence of comorbidities. The APACHE-IV scores were calculated automatically using data from the first 24 h of ICU admission. Comorbidities present at admission were identified using the ICD-9.

### Computation and Propensity Score Matching

Baseline characteristics included age, sex, BMI, disease severity scores, ethnicity, comorbidities, ICU type, and treatment. To reduce confounding from differences in baseline characteristics, 1:1 propensity score (PS) matching was performed using “nearest neighbor” matching, with a caliper width of 0.01 across age, sex, BMI, APACHE-IV score, APS-II, and cardiovascular comorbidities (arrhythmias, coronary artery bypass grafting [CABG], congestive heart failure [CHF], hypertension, remote myocardial infarction [RMI], presence of a pacemaker, procedural coronary intervention (PCI) 6 months prior to admission, and valve disease). As inotropes were preferred in patients with a lower cardiac index (CI), the clinical outcomes were compared between two groups after 1:1 PS matching across CI.

### Statistical Analysis

Categorical variables were reported as numbers (%) and compared between groups using the Pearson chi-square test. Continuous variables were reported as the mean (± standard deviation [SD]) and compared between two and more groups using the Student *t*-test and one-way analysis of variance, respectively. The influence of the use of different kinds of inotropes on ICU and hospital mortality was determined using logistic multivariate regression analysis. Kaplan-Meier survival curves (log rank method) were used to compare the difference in survival between the inotrope and non-inotrope groups for ICU and hospital stays. All statistical analyses were performed using Navicat for PostgreSQL (11.2.9), IBM® SPSS® Statistics 26, or SigmaPlot 13.0. All tests were two-tailed, and *p* < 0.05 was considered statistically significant.

## Results

**Figure 1:**
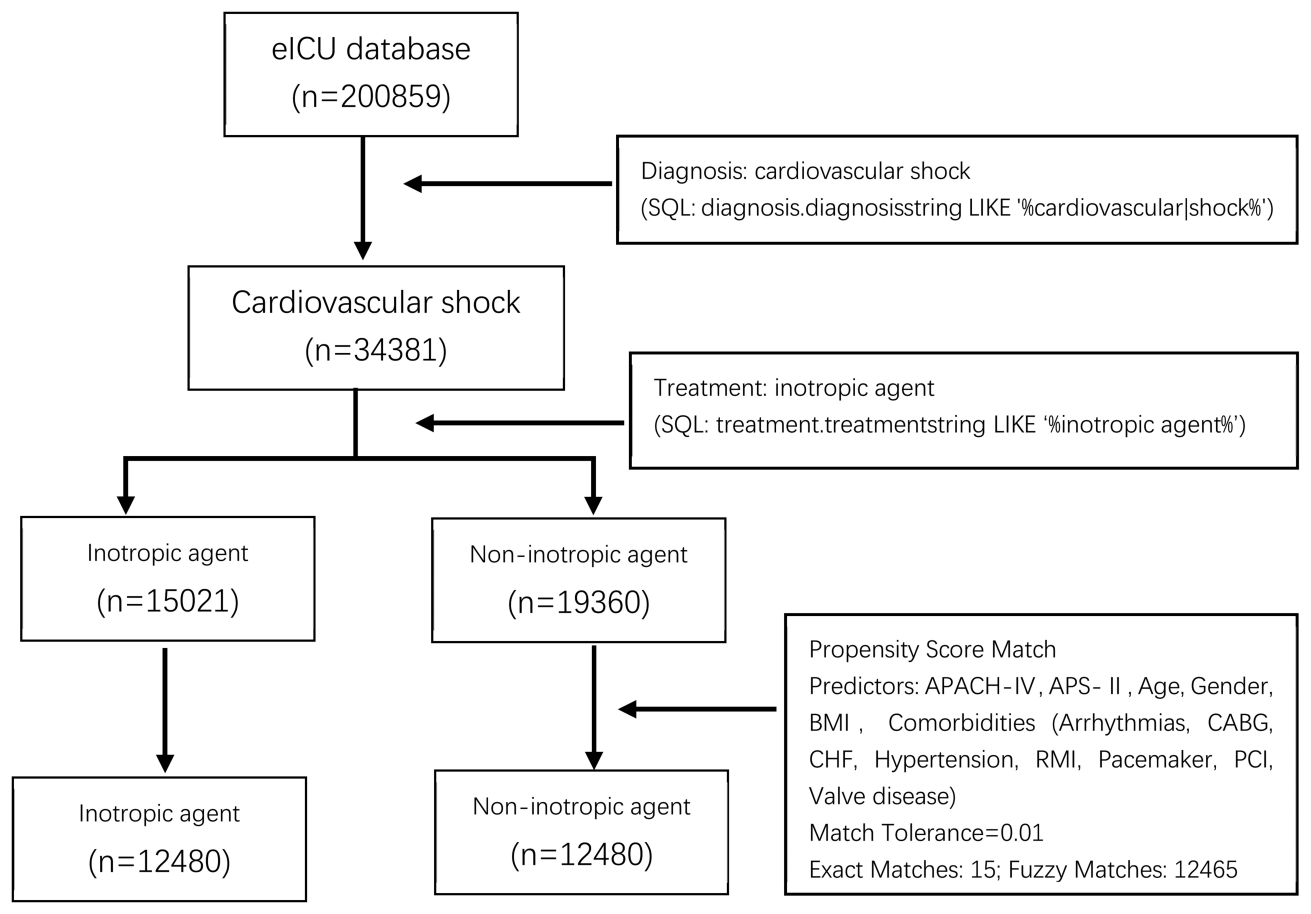
Process of selecting the study subjects. eICU, The Philips Electronic ICU; SQL, Structured Query Language; BMI, Body Mass Index; APACH-IV, acute physiology and chronic health evaluation score IV; APS-II, simplified acute physiology score II; CABG, Coronary Artery Bypass Grafting; CHF, congestive heart failure; RMI, Remote Myocardial Infarction; PCI, Procedural Coronary Intervention.

Of the 34,381 patients diagnosed with CS, 15,021 (43.69%) were administered inotropes during hospitalization. Figure 1 shows the patient inclusion flowchart. There was a significant difference in age, sex, BMI, disease severity scores, ethnicity, and comorbidities between the groups (all *p* < 0.05). Baseline characteristics of all patients are summarized in Table 1.

**Table 1:** Baseline characteristics of before and after propensity score matching between patients receiving inotropic agent and patients not receiving inotropic agent.

	**Before PSM**			**After PSM**		
	**Inotropic (*n* = 15,021)**	**Non-inotropic (*n* = 19,360)**	***P*-value**	**Inotropic (*n* = 12,480)**	**Non-inotropic (*n* = 12,480)**	***P*-value**
Age, yrs	66.47 ± 14.93	64.45 ± 16.76	<0.001	66.20 ± 14.97	66.37 ± 16.21	0.363
Male, *n* (%)	8,011 (53.33)	9,970 (51.50)	<0.001	6,567 (52.62)	6,508 (52.15)	0.455
BMI (kg/m^2^)	28.28 ± 7.13	27.89 ± 7.11	<0.001	28.98 ± 8.57	29.12 ± 8.92	0.216
APACHE-IV	79.82 ± 31.24	60.59 ± 23.96	<0.001	68.70 ± 23.58	68.50 ± 23.29	0.51
APS-II	66.15 ± 30.25	47.73 ± 21.96	<0.001	54.75 ± 22.20	53.15 ± 21.69	<0.01
Ethnicity, *n* (%)						
Caucasian	11,645 (77.52)	14,704 (75.95)	<0.001	9,731 (77.97)	9,446 (75.69)	<0.01
African American	1,384 (9.21)	2,142 (11.06)	<0.01	1,093 (8.76)	1,384 (11.09)	<0.01
Native American	141 (0.94)	168 (0.87)	0.12	115 (0.92)	141 (1.13)	0.1
Asian	223 (1.48)	420 (2.17)	<0.01	198 (1.59)	229 (1.83)	0.13
Hispanic	763 (5.08)	798 (4.12)	0.36	630 (5.05)	602 (4.82)	0.41
Comorbidities, *n* (%)						
Hypertension	7,822 (52.07)	9,768 (50.45)	<0.001	6,512 (52.18)	6,227 (49.90)	<0.001
CHF	3,235 (21.54)	3,219 (16.63)	<0.001	2,547 (20.41)	2,529 (20.26)	0.778
RMI	1,590 (10.59)	1,692 (8.74)	<0.001	1,250 (10.02)	1,303 (10.44)	0.268
Valve disease	892 (5.94)	777 (4.01)	<0.001	679 (5.44)	639 (5.12)	0.258
CABG	1,070 (7.12)	1,121 (5.79)	<0.001	844 (6.76)	821 (6.58)	0.560
PCI (within 2 years)	1,021 (6.80)	1,129 (5.83)	<0.001	814 (6.52)	823 (6.59)	0.818
Arrhythmias	2,510 (16.71)	2,446 (12.63)	<0.001	1,958 (15.69)	1,867 (14.96)	0.110
Pacemaker	701 (4.67)	655 (3.38)	<0.001	530 (4.25)	521 (4.17)	0.777
COPD	2,626 (17.48)	3,147 (16.26)	0.003	2,183 (17.49)	2,026 (16.23)	0.008
Renal failure	1,490 (9.92)	1,688 (8.72)	<0.001	1,192 (9.55)	1,262 (10.11)	0.137
Stroke	1,489 (9.91)	1,862 (9.62)	0.36	1,195 (9.58)	1,307 (10.47)	0.018
Diabetes	2,281 (15.19)	3,104 (16.03)	0.03	4,017 (32.19)	4,295 (34.42)	<0.001
Cancer	2,703 (17.99)	3,319 (17.14)	0.04	2,215 (17.75)	2,242 (17.96)	0.655
Infectious disease	367 (2.44)	509 (2.63)	0.28	306 (2.45)	338 (2.71)	0.201
Rheumatoid arthritis	367 (2.44)	377 (1.95)	<0.001	317 (2.54)	230 (1.84)	<0.001
Invasive ventilation, *n* (%)	4,635 (30.86)	2,224 (11.49)	<0.001	3,033 (24.30)	1,821 (14.59)	<0.001
Non-invasive ventilation, *n* (%)	2,204 (14.67)	2,194 (11.33)	<0.001	1,847 (14.80)	1,489 (11.93)	<0.001

*The predictors for matching were age, gender, BMI, APACHE-IV, APS-II, and comorbidities. Data displayed as n (%) or mean ± standard deviation, with P-value for between-groups comparison using chi squared or analysis of variance. PSM, propensity score matching; BMI, Body Mass Index; APACH-IV, acute physiology and chronic health evaluation score IV; APS-II, simplified acute physiology score II; CHF, congestive heart failure; RMI, Remote Myocardial Infarction; CABG, Coronary Artery Bypass Grafting; PCI, Procedural Coronary Intervention; COPD, chronic obstructive pulmonary disease*.

The final PS-matched cohort involved 12,480 patients in each group. There were no significant differences in the APACHE-IV score, age, sex, BMI, and cardiovascular comorbidities between the groups after PS matching. The inotrope group included a higher percentage of patients with hypertension (52.18 vs. 49.90%, *p* < 0.001), chronic obstructive pulmonary disease (17.49 vs. 16.23%, *p* = 0.008), and rheumatoid arthritis (2.54 vs. 1.84%, *p* < 0.001), whereas the non-inotrope group included more patients with stroke (9.58 vs. 10.47%, *p* = 0.018) and diabetes (32.19 vs. 34.42%, *p* < 0.001). The APS-II scores were slightly higher in the inotrope group than in the non-inotrope group (54.75 ± 22.20 vs. 53.15 ± 21.69, *p* < 0.001) after PS matching. More patients in the inotrope group received supportive treatments, including invasive ventilation (24.30 vs. 14.59%, *p* < 0.001) and non-invasive ventilation (14.80 vs. 11.93%, *p* < 0.001). The other unmatched baseline characteristics included ethnicity (Table 1).

To determine the risk factors of ICU and hospital mortality in CS patients, multivariate logistic regression analysis was performed. Age (per 10-year increase) (hazard ratio [HR] 1.084, 95% confidence interval [CI]: 1.050–1.118, *p* < 0.001), BMI (high vs. low) (HR 1.243, 95% CI: 1.043–1.481, *p* = 0.015), APACHE-IV (per 10-point increase) (HR 1.303, 95% CI: 1.280–1.327, *p* < 0.001), inotropic agent (HR 3.248, 95% CI: 2.950–3.578, *p* < 0.001), and ventilation days (HR 1.045, 95% CI: 1.032–1.059, *p* < 0.001) were associated with higher ICU mortality. Moreover, risk factor for patients with increased hospital mortality included age (per 10-year increase) (HR 1.141, 95% CI: 1.110–1.172, *p* < 0.001), sex (female) (HR 1.096, 95% CI: 1.022–1.176, *p* = 0.008), BMI (high vs. low) (HR 1.276, 95% CI: 1.099–1.483, *p* = 0.002), APACHE-IV (per 10-points increase) (HR 1.260, 95% CI: 1.241–1.280, *p* < 0.001), inotropic agent (HR 2.458, 95% CI: 2.275–2.655, *p* < 0.001), and ventilation days (HR 1.038, 95% CI: 1.027–1.049, *p* < 0.001) (Table 2).

**Table 2:** Regression analysis was performed to investigate the risk factors of ICU mortality and hospital mortality for cardiogenic shock patients.

	**ICU mortality**		**Hospital mortality**	
	**HR [95%CI]**	***P*-value**	**HR [95%CI]**	***P*-value**
Age (per 10-year increase)	1.084 [1.050, 1.118]	<0.001	1.141 [1.110, 1.172]	<0.001
Gender (Female)	1.055 [0.971, 1.146]	0.204	1.096 [1.022, 1.176]	0.008
BMI (Normal vs. LOW)	0.915 [0.836, 1.001]	0.052	0.859 [0.796, 0.927]	<0.001
BMI (High vs. LOW)	1.243 [1.043,1.481]	0.015	1.276[[1.099,1.483]	0.002
APACHE-IV (per 10-points increase)	1.303 [1.280, 1.327]	<0.001	1.260 [1.241, 1.280]	<0.001
Inotropic agent	3.248 [2.950, 3.578]	<0.001	2.458 [2.275, 2.655]	<0.001
ICU LOS (hours)	0.999 [0.999, 0.999]	<0.001	0.999 [0.999,1.000]	0.002
Invasive ventilation	0.860 [0.767, 0.965]	0.010	0.764 [0.683, 0.856]	<0.001
Non-invasive ventilation	1.023 [0.788, 1.328]	0.863	1.045 [0.925, 1.182]	0.478
Ventilation days	1.045 [1.032, 1.059]	<0.001	1.038 [1.027, 1.049]	<0.001

*Data displayed as HR [95%CI] with P-value using multivariate logistic regression using stepwise backward variable selection. BMI, Body Mass Index; APACH-IV, acute physiology and chronic health evaluation score IV; LOS, length of stay; HR, hazard ratio; 95%CI, 95% confidence interval*.

**Figure 2:**
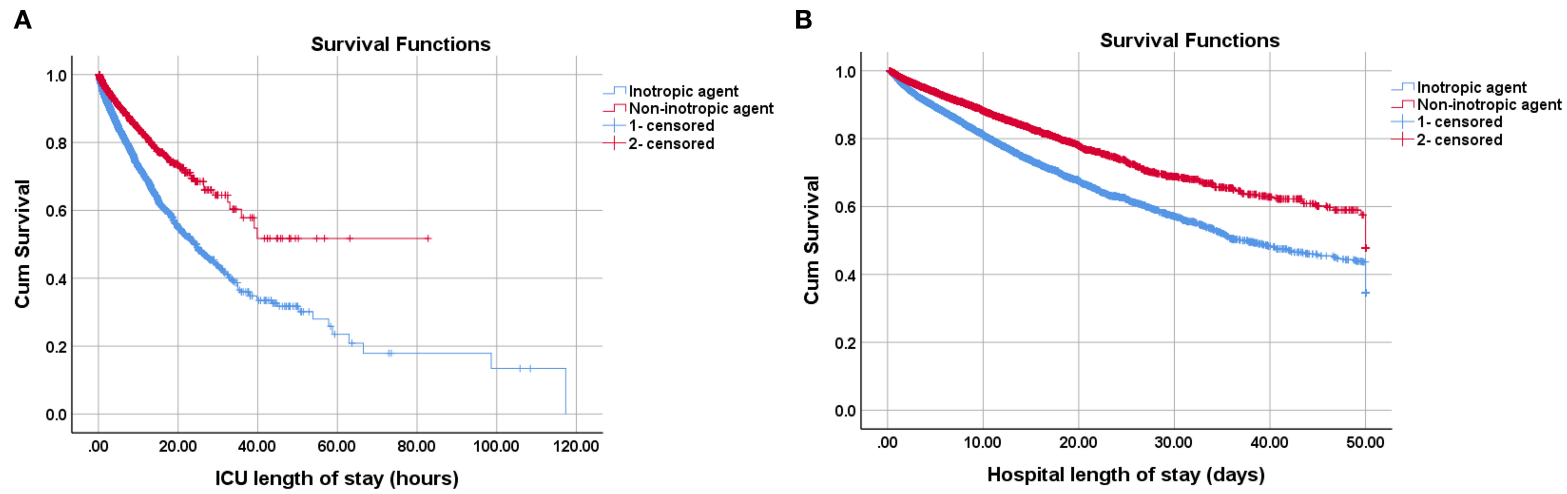
Kaplan-Meier survival curves of cardiogenic shock patients receiving inotropic agents or not on **(A)** ICU and **(B)** hospital. The average survival hours of inotropic group and non-inotropic group in the ICU were 39.37 ± 2.90 and 51.95 ± 2.94 h, *P* < 0.001; The average survival days of inotropic group and non-inotropic group in the hospital were 32.37 ± 0.31 and 37.61 ± 0.36 d, *P* < 0.001. The survival curves were performed by Log Rank method.

Given that inotrope use was an independent risk factor for both ICU and hospital mortality, we investigate the effect of inotrope use to clinical outcome. The comparison of clinical outcomes before and after PS matching between the two groups is shown in Table 3. Patients receiving inotropic agents were at higher risk of both ICU (17.24 vs. 6.08%, unadjusted odds ratio [OR]: 3.22, 95% CI: 2.95–3.51, *p* < 0.001) and hospital mortality (24.03 vs. 12.4%, unadjusted OR: 2.24, 95% CI: 2.09–2.39, *p* < 0.001). The inotrope group had a significantly longer ICU LOS (124.13 ± 155.63 vs. 79.07 ± 120.86 h, MD: 45.06, 95% CI: 41.60–48.52, *p* < 0.001), hospital LOS (11.64 ± 10.28 days vs. 10.23 ± 9.28 days, MD: 1.41, 95% CI: 1.17–1.65, *p* < 0.001), and duration of ventilation (5.33 ± 5.44 vs. 4.34 ± 4.48 h, MD: 0.99, 95% CI: 0.87–1.11, *p* < 0.001) than the non-inotrope group. The ARF rate was also higher in the inotrope group (32.17 vs. 24.39%, unadjusted OR: 1.47, 95% CI: 1.39–1.55, *p* < 0.001). The average length of stay hours of the inotrope and non-inotrope groups in ICU were 39.37 ± 2.90 h and 51.95 ± 2.94 h, respectively (*p* < 0.001). The average survival days of the inotrope and non-inotrope groups in the hospital were 32.37 ± 0.31 and 37.61 ± 0.36 days, respectively (*p* < 0.001) (Figure 2).

**Table 3:** Clinical outcomes compared before and after propensity score matching between patients receiving inotropic agent and patients not receiving inotropic agent.

	**Before PSM**				**After PSM**			
	**Inotropic** ** (*n* = 15,021)**	**Non-inotropic** **(*n* = 19,360)**	**MD/OR [95%CI]**	***P*-value**	**Inotropic** ** (*n* = 12,480)**	**Non-inotropic** ** (*n* = 12,480)**	**MD/OR [95%CI]**	***P*-value**
ARF, *n* (%)	5,228 (34.80)	4,391 (22.68)	1.82 [1.74, 1.91]	<0.001	4,015 (32.17)	3,044 (24.39)	1.47 [1.39, 1.55]	<0.001
Ventilation LOS (days)	5.31 ± 5.43	4.21 ± 4.42	1.10 [0.99, 1.21]	<0.001	5.33 ± 5.44	4.34 ± 4.48	0.99 [0.87, 1.11]	<0.001
ICU mortality, *n* (%)	3,294 (21.93)	996 (5.14)	5.18 [4.81, 5.58]	<0.001	2,152 (17.24)	759 (6.08)	3.22 [2.95, 3.51]	<0.001
ICU LOS (hours)	126.48 ± 157.85	74.89 ± 119.50	51.59 [48.56, 54.62]	<0.001	124.13 ± 155.63	79.07 ± 120.86	45.06 [41.60, 48.52]	<0.001
Hospital LOS (days)	11.44 ± 10.36	9.58 ± 8.96	1.86 [1.65, 2.07]	<0.001	11.64 ± 10.28	10.23 ± 9.28	1.41 [1.17, 1.65]	<0.001
Hospital mortality, *n* (%)	4,371 (29.10)	2,034 (10.51)	3.50 [3.30, 3.70]	<0.001	2,999 (24.03)	1,547 (12.40)	2.24 [2.09, 2.39]	<0.001

*Data displayed as N (%) or mean ± standard deviation, and mean difference or odds ratio [95% confidence interval] with P-value for between-groups comparison using chi squared or analysis of variance. PSM, propensity score matching; ARF, acute renal failure; LOS, length of stay; MD, mean difference; OR, odds ratio; HR, hazard ratio; 95%CI, 95% confidence interval*.

Inotropes were preferred in patients with lower CI, the clinical outcomes were compared between two groups after 1:1 PS matching across CI. After PS matching, 359 patients for each group were included, the risk of ICU (OR 5.65, 95% CI, 3.17–10.08, *p* < 0.001) and hospital (OR 2.63, 95% CI: 1.75–3.95, *p* < 0.001) mortality in the inotrope group was significantly higher (Table 4). We also investigated the correlation between different types of inotropes and ICU and hospital mortality. Among patients receiving inotrope agents, those receiving dobutamine (HR: 0.882, 95% CI: 0.820–0.949, *p* < 0.001), LD epinephrine (HR: 0.763, 95% CI: 0.689–0.843, *p* < 0.001), HD epinephrine (HR: 0.818, 95% CI: 0.748–0.895, *p* < 0.001), and LD norepinephrine (HR: 0.873, 95% CI: 0.838–0.910, *p* < 0.001) had lower risk of ICU mortality. MD (HR: 1.757, 95% CI: 1.137–2.714, *p* = 0.011) and HD (HR: 2.454, 95% CI: 1.732–3.478, *p* < 0.001) dopamine increased the risk of ICU mortality, while dobutamine (HR: 1.758, 95% CI: 1.467–2.108, *p* < 0.001), HD dopamine (HR: 1.772, 95% CI: 1.158–2.712, *p* = 0.008), LD epinephrine (HR: 1.906, 95% CI: 1.318–2.755, *p* < 0.001), HD epinephrine (HR: 3.120, 95% CI: 2.506–3.883, *p* < 0.001), and HD norepinephrine (HR: 1.405, 95% CI: 1.263–1.563, *p* < 0.001) significantly increased the risk of hospital mortality. In contrast, milrinone (HR: 0.559, 95% CI: 0.430–0.727, *p* < 0.001) significantly decreased the risk of hospital mortality. The results are shown in Table 5. Baseline characteristics of the different inotrope groups are shown in Supplementary Table 1.

**Table 4:** Clinical outcomes after propensity score matched by cardiac index between inotrope group and non-inotrope group.

	**Inotropic** ** (*n* = 359)**	**Non-inotropic (*n* = 359)**	**MD/OR [95%CI]**	***P*-value**
ARF, *n* (%)	120 (33.43)	102 (28.41)	1.27 [0.92, 1.74]	0.15
Ventilation LOS (days)	4.22 ± 5.60	4.04 ± 5.31	0.18 [−0.62, 0.98]	0.66
ICU mortality, n (%)	71 (19.78)	15 (4.18)	5.65 [3.17, 10.08]	<0.001
ICU LOS (hours)	180.16 ± 244.22	162.05 ± 233.43	18.11 [−16.84, 53.06]	0.31
Hospital mortality, *n* (%)	89 (24.79)	40 (11.14)	2.63 [1.75, 3.95]	<0.001
Hospital LOS (days)	13.77 ± 11.09	13.83 ± 11.80	−0.06 [−1.74, 1.62]	0.94

*Data displayed as n (%) or mean ± standard deviation, and mean difference or odds ratio [95% confidence interval] with P-value for between-groups comparison using chi squared or analysis of variance. PSM, propensity score matching; ARF, acute renal failure; LOS, length of stay; MD, mean difference; OR, odds ratio; HR, hazard ratio; 95%CI, 95% confidence interval*.

**Table 5:** ICU and hospital mortality risk analysis in the patients with cardiogenic shock receiving different inotropic agents.

**(Expired as reference category)**	**ICU discharge**		**Hospital discharge**	
	**HR [95%CI]**	***P*-value**	**HR [95%CI]**	***P*-value**
Dobutamine	0.882 [0.820, 0.949]	<0.001	1.758 [1.467, 2.108]	<0.001
Dopamine ≤ 5 μg/kg/min	0.911 [0.718, 1.155]	0.442	0.781 [0.447, 1.367]	0.387
Dopamine 5–15 μg/kg/min	1.757 [1.137, 2.714]	0.011	0.875 [0.707, 1.082]	0.218
Dopamine > 15 μg/kg/min	2.454 [1.732, 3.478]	<0.001	1.772 [1.158, 2.712]	0.008
Epinephrine ≤ 0.1 μg/kg/min	0.763 [0.689, 0.843]	<0.001	1.906 [1.318, 2.755]	0.001
Epinephrine > 0.1 μg/kg/min	0.818 [0.748, 0.895]	<0.001	3.120 [2.506, 3.883]	<0.001
Norepinephrine ≤ 0.1 μg/kg/min	0.873 [0.838, 0.910]	0.001	0.943 [0.824, 1.079]	0.393
Norepinephrine > 0.1 μg/kg/min	0.993 [0.949, 1.038]	0.749	1.405 [1.263, 1.563]	<0.001
Milrinone	1.068 [0.964, 1.184]	0.208	0.559 [0.430, 0.727]	<0.001

*Data displayed as HR [95%CI] with P-value using multivariate logistic regression using stepwise backward variable selection. HR, hazard ratio; 95%CI, 95% confidence interval*.

## Discussion

Inotropes are a double-edged sword; while they improve cardiac output, they also bring about side effects, including arrhythmias, myocardial ischemia, and in some cases, hypotension (1, 3, 4). This study found that the use of inotropes was associated with a higher risk of hospital mortality. Among inotropes, norepinephrine and milrinone were relatively safe at LDs. To our knowledge, this is the largest study using a national dataset to assess the usefulness of inotropes in CS. Patients with worse general conditions may have a higher risk of hospital mortality. We attempted to account for this by controlling for the APACHE-IV score; age; BMI; sex; and history of cardiovascular comorbidities, including arrhythmias, CABG, CHF, hypertension, RMI, presence of a pacemaker, PCI, and valve disease. Despite controlling for these factors, the results still showed that inotrope use was associated with unfavorable outcomes in terms of ICU mortality, hospital mortality, and ARF. Moreover, the average survival time of the inotropic group was significantly shorter than that of the non-inotropic group. These findings are consistent with those of previous studies (12–14). Kalogeropoulos et al. examined the association of in-hospital inotrope use with 6-month outcomes in the ESCAPE trial and found that in the absence of CS or end-organ hypoperfusion, inotrope use during hospitalization for heart failure is associated with unfavorable 6-month outcomes, regardless of admission SBP, CI, or heart failure etiology (13).

Norepinephrine, a very potent and reliable vasopressor, increases mean arterial pressure without any concomitant increase in heart rate. Norepinephrine and epinephrine are currently the most commonly used inotropic agents in CS (15–17). In the present study, norepinephrine at a dose of < 0.1 μg/kg/min did not increase the risk of hospital mortality but reduced the risk of ICU mortality. Meanwhile, although epinephrine decreased the risk of ICU mortality, it also significantly increased the risk of hospital mortality. These findings are consistent with those of previous studies that indicated that adrenaline use was associated with marked worsening in cardiac and renal biomarkers (17). However, some early studies comparing epinephrine and norepinephrine in patients with septic shock found no significant differences in the outcome (18). Despite these findings, some clinicians believe that these drugs may have specific effects that could influence the outcomes of CS patients. Accordingly, several clinical trials and outcome studies have compared the usefulness of epinephrine with that of norepinephrine in CS and found that epinephrine was associated with a higher incidence of refractory shock and short-term mortality (19, 20).

A multicentre randomized trial showed that dopamine is associated with a higher 28-day mortality than norepinephrine (5). Meanwhile, another meta-analysis revealed that norepinephrine is associated with a lower 28-day mortality, lower risk of arrhythmic events, and fewer gastrointestinal reactions compared with dopamine (21). The results of this study showed that dopamine at a dose of >5 μg/kg/min was associated with a higher risk of ICU mortality, and the risk of hospital mortality was also significantly increased when dopamine was used at a dose of >15 μg/kg/min. Collectively, the findings of this study and those of previous studies indicate that HD dopamine should be used with caution in CS.

As a pure inotrope, dobutamine is a predominantly beta-1-adrenergic agonist with weak beta-2 and alpha-1 activity. Dobutamine has been recommended as a first-line inotrope based on clinical experience (7, 8). In this study, we found that dobutamine significantly decreased ICU mortality but also increased hospital mortality. These results indicate that dobutamine can be recommended in ICU patients, but its administration should be accompanied by vital sign or hemodynamic monitoring. However, its long-term effects and our findings highlight the need for further targeted research.

Inotropic agents can be divided into adrenergic and noradrenergic inotropic agents (22). In this study, we observed that the phosphodiesterase antagonist milrinone, a type of noradrenergic inotropic agent, significantly decreased the risk of hospital mortality. Milrinone is a positive inotropic agent and a peripheral vasodilator. It can be administered intravenously to patients with advanced systolic heart failure to improve cardiac performance. We also observed that patients in the milrinone subgroup had lower APACHE-IV scores (Supplementary Table 1), and the primary disease resulting in CS was different from that found in other subgroups. These unbalanced baseline characteristics may have influenced the results.

Our study has some limitations. First, this was a retrospective observational study, and although we adjusted for several clinical covariates in the PS matching analysis, potential biases may exist due to unmatched and unmeasured confounding factors. Second, we did not analyse hemodynamic parameters, such as invasive blood pressure and central venous pressure, because these data were not available in the eICU database. However, we did compare the clinical outcomes after PS matching according to the CI, and the matched analysis showed that inotrope use was still associated with a higher risk of mortality. Similarly, diagnoses were identified using the ICD-9 codes. The data were collected at different centers, and there were no standard criteria for data collection, which may have led to confounding. Third, we did not have data on long-term follow-up; thus, we were not able to evaluate the association between inotrope use and the long-term survival of CS patients. Randomized controlled trials are required to validate our findings.

In conclusion, inotropes should be used with caution in CS, and should be employed at the lowest dose and for the shortest span possible since our results support the view that inotrope use is associated with a higher risk of mortality. LD norepinephrine and milrinone are associated with lower risk of hospital mortality in these patients. When large doses of inotropes are needed, cardiac assistance and other supportive treatments should be initiated early in the course of treatment.

## Data Availability Statement

The datasets presented in this study can be found in online repositories. The names of the repository/repositories and accession number(s) can be found in the article/Supplementary Material.

## Ethics Statement

The studies involving human participants were reviewed and approved by Research Ethics Committee of Wuxi People's Hospital. Written informed consent for participation was not required for this study in accordance with the national legislation and the institutional requirements.

## Author Contributions

FG was responsible for data validation and the writing of the manuscript and YZ was responsible for research design, data extraction, and analysis. All authors contributed to the article and approved the submitted version.

## Funding

This work was funded by the Top Talent Support Program for young and middle-aged people of the Wuxi Health Committee (project number: BJ2020007).

## Conflict of Interest

The authors declare that the research was conducted in the absence of any commercial or financial relationships that could be construed as a potential conflict of interest.

## Publisher's Note

All claims expressed in this article are solely those of the authors and do not necessarily represent those of their affiliated organizations, or those of the publisher, the editors and the reviewers. Any product that may be evaluated in this article, or claim that may be made by its manufacturer, is not guaranteed or endorsed by the publisher.
