# Supplementary material for: Inotrope Use and Intensive Care Unit Mortality in Patients With Cardiogenic Shock: An Analysis of a Large Electronic Intensive Care Unit Database
Source: Front Cardiovasc Med. 2021 Sep 21;8:696138. doi: 10.3389/fcvm.2021.696138 (PMC8490645; doi:10.3389/fcvm.2021.696138)
Supplement: Supplementary file 1 [file Table_1.DOCX]

Supplementary Material

Table S.Clinical characteristic of different inotropic agents.

|  | **Dopamine**  **≤5mg/kg/min** | **Dopamine**  **5-15mg/kg/min** | **Dopamine**  **>15mg/kg/min** | **Dobutamine** | **Epinephrine**  **≤0.1mg/kg/min** | **Epinephrine**  **>0.1mg/kg/min** | **Norepinephrine**  **≤0.1mg/kg/min** | **Norepinephrine**  **>0.1mg/kg/min** | **Milrinone** |
| --- | --- | --- | --- | --- | --- | --- | --- | --- | --- |
| N | 133 | 510 | 112 | 558 | 316 | 432 | 2260 | 2615 | 261 |
| Age(years) | 69.41±13.60 | 68.77±13.83 | 67.37±13.27 | 67.31±14.49 | 64.35±14.85 | 64.37±14.80 | 65.93±14.71 | 65.88±15.26 | 64.82±13.18 |
| Male (%) | 74(55.64) | 268(52.55) | 63(56.25) | 344(61.65) | 193(61.08) | 236(54.63) | 1227(54.29) | 1378(52.70) | 163(62.45) |
| BMI (kg/m2) | 30.35±8.09 | 29.06±8.49 | 30.69±8.03 | 29.13±8.33 | 29.50±8.62 | 29.80±8.78 | 29.61±8.94 | 29.05±9.10 | 29.28±6.99 |
| APACHE-Ⅳ | 61.65±23.21 | 67.74±23.73 | 80.78±28.24 | 72.26±25.69 | 76.81±31.38 | 84.69±31.67 | 71.03±23.85 | 75.40±25.52 | 68.34±26.29 |
| APS-II | 47.251±19.68 | 53.36±22.17 | 65.79±25.73 | 58.91±24.21 | 64.58±30.23 | 71.47±30.09 | 57.39±22.32 | 61.51±23.94 | 56.42±24.67 |
| **Ethnicity, n (%)** |  |  |  |  |  |  |  |  |  |
| Caucasian | 115(86.47) | 399(78.24) | 92(82.14) | 446(79.93) | 257(81.33) | 358(82.87) | 1853(81.99) | 2044(78.16) | 201(77.01) |
| African American | 6(4.51) | 39(7.65) | 8(7.14) | 49(8.78) | 20(6.33) | 26(6.02) | 134(5.93) | 198(7.57) | 25(9.58) |
| Native American | 0(0.00) | 7(1.37) | 2(1.79) | 5(0.90) | 7(2.22) | 12(2.78) | 27(1.19) | 39(1.49) | 2(0.77) |
| Asian | 2(1.50) | 4(0.78) | 1(0.89) | 5(0.90) | 5(1.58) | 3(0.69) | 22(0.97) | 26(0.99) | 5(1.92) |
| Hispanic | 4(3.01) | 34(6.67) | 5(4.46) | 23(4.12) | 18(5.70) | 23(5.32) | 101(4.47) | 161(6.16) | 16(6.13) |
| Other/Unknown | 6(4.51) | 27(5.29) | 4(3.57) | 30(5.38) | 9(2.85) | 10(2.31) | 123(5.44) | 147(5.62) | 12(4.60) |
| **Comorbidities, n (%)** |  |  |  |  |  |  |  |  |  |
| CVD | 116(87.22) | 379(74.31) | 84(75.00) | 431(77.24) | 162(51.27) | 197(45.60) | 1521(67.30) | 1671(63.90) | 223(85.44) |
| Hypertension | 85(63.91) | 287(56.27) | 61(54.46) | 289(51.79) | 113(35.76) | 146(33.80) | 1122(49.65) | 1243(47.53) | 139(53.26) |
| CHF | 55(41.35) | 142(27.84) | 33(29.46) | 222(39.78) | 48(15.19) | 54(12.50) | 473(20.93) | 485(18.55) | 109(41.76) |
| RMI | 30(22.56) | 80(15.69) | 12(10.71) | 111(19.89) | 33(10.44) | 33(7.64) | 269(11.90) | 277(10.59) | 62(23.75) |
| Valve disease | 16(12.03) | 46(9.02) | 13(11.61) | 58(10.39) | 36(11.39) | 27(6.25) | 146(6.46) | 122(4.67) | 46(17.62) |
| CABG | 17(12.78) | 49(9.61) | 14(12.50) | 66(11.83) | 14(4.43) | 15(3.47) | 134(5.93) | 148(5.66) | 25(9.58) |
| PCI | 22(16.54) | 57(11.18) | 9(8.04) | 67(12.01) | 21(6.65) | 24(5.56) | 154(6.81) | 147(5.62) | 48(18.39) |
| Arrhythmias | 41(30.83) | 112(21.96) | 18(16.07) | 129(23.12) | 39(12.34) | 45(10.42) | 325(14.38) | 368(14.07) | 66(25.29) |
| Pacemaker | 9(6.77) | 26(5.10) | 5(4.46) | 61(10.93) | 8(2.53) | 14(3.24) | 97(4.29) | 111(4.24) | 16(6.13) |
| Renal Failure | 12(9.02) | 44(8.63) | 12(10.71) | 45(8.06) | 28(8.86) | 38(8.80) | 216(9.56) | 270(10.33) | 21(8.05) |
| COPD | 28(21.05) | 89(17.45) | 14(12.50) | 102(18.28) | 32(10.13) | 39(9.03) | 398(17.61) | 425(16.25) | 44(16.86) |
| Rheumatoid Arthritis | 3(2.26) | 12(2.35) | 4(3.57) | 12(2.15) | 5(1.58) | 6(1.39) | 56(2.48) | 77(2.94) | 9(3.45) |
| Stroke | 14(10.53) | 51(10.00) | 14(12.50) | 61(10.93) | 16(5.06) | 23(5.32) | 183(8.10) | 236(9.02) | 21(8.05) |
| Infectious Disease | 5(3.76) | 6(1.18) | 2(1.79) | 9(1.61) | 3(0.95) | 7(1.62) | 53(2.35) | 73(2.79) | 5(1.92) |
| Cancer | 19(14.29) | 73(14.31) | 21(18.75) | 69(12.37) | 33(10.44) | 50(11.57) | 425(18.81) | 504(19.27) | 22(8.43) |
| Diabetes | 50(37.59) | 168(32.94) | 48(42.86) | 184(32.97) | 78(24.68) | 87(20.14) | 750(33.19) | 822(31.43) | 86(32.95) |
| **ICU type, n (%)** |  |  |  |  |  |  |  |  |  |
| Med-Surg ICU | 56(42.11) | 239(46.86) | 61(54.46) | 308(55.20) | 101(31.96) | 167(38.66) | 1281(56.68) | 1553(59.39) | 78(29.89) |
| MICU | 14(10.53) | 58(11.37) | 8(7.14) | 54(9.68) | 28(8.86) | 57(13.19) | 291(12.88) | 344(13.15) | 12(4.60) |
| Cardiac ICU | 9(6.77) | 39(7.65) | 7(6.25) | 39(6.99) | 10(3.16) | 22(5.09) | 96(4.25) | 120(4.59) | 24(9.20) |
| CCU-CTICU | 34(25.56) | 75(14.71) | 11(9.82) | 79(14.16) | 71(22.47) | 38(8.80) | 288(12.74) | 154(5.89) | 69(26.44) |
| CSICU | 3(2.26) | 47(9.22) | 14(12.50) | 38(6.81) | 58(18.35) | 66(15.28) | 130(5.75) | 195(7.46) | 45(17.24) |
| SICU | 12(9.02) | 30(5.88) | 7(6.25) | 23(4.12) | 24(7.59) | 48(11.11) | 107(4.73) | 143(5.47) | 24(9.20) |
| Neuro ICU | 3(2.26) | 16(3.14) | 3(2.68) | 8(1.43) | 0(0.00) | 8(1.85) | 48(2.12) | 63(2.41) | 1(0.38) |
| CTICU | 2(1.50) | 6(1.18) | 0(0.00) | 9(1.61) | 24(7.59) | 26(6.02) | 19(0.84) | 43(1.64) | 8(3.07) |
| **Treatment, n(%)** |  |  |  |  |  |  |  |  |  |
| Invasive Ventilation | 37(27.82) | 116(22.75) | 32(28.57) | 173(31.00) | 171(54.11) | 146(33.80) | 684(30.27) | 801(30.63) | 121(46.36) |
| Non-invasive ventilation | 15(11.28) | 60(11.76) | 24(21.43) | 89(15.95) | 53(16.77) | 48(11.11) | 346(15.31) | 385(14.72) | 28(10.73) |
| Dialysis | 4(3.01) | 23(4.51) | 8(7.14) | 16(2.87) | 24(7.59) | 24(5.56) | 154(6.81) | 192(7.34) | 9(3.45) |
| Cardiac angiography | 14(10.53) | 40(7.84) | 6(5.36) | 52(9.32) | 18(5.70) | 15(3.47) | 41(1.81) | 54(2.07) | 33(12.64) |
| Stent placement | 10(7.52) | 20(3.92) | 4(3.57) | 12(2.15) | 5(1.58) | 4(0.93) | 13(0.58) | 17(0.65) | 5(1.92) |
| Defibrillation | 0(0.00) | 2(0.39) | 2(1.79) | 1(0.18) | 0(0.00) | 4(0.93) | 1(0.04) | 4(0.15) | 1(0.38) |
| Pacemaker | 10(7.52) | 30(5.88) | 11(9.82) | 19(3.41) | 22(6.96) | 12(2.78) | 28(1.24) | 31(1.19) | 14(5.36) |
| PAC | 4(3.01) | 6(1.18) | 0(0.00) | 17(3.05) | 18(5.70) | 5(1.16) | 123(5.44) | 12(0.46) | 40(15.33) |
| IABP | 18(13.53) | 43(8.43) | 6(5.36) | 61(10.93) | 41(12.97) | 35(8.10) | 68(3.01) | 65(2.49) | 67(25.67) |
| CABG | 8(6.02) | 10(1.96) | 0(0.00) | 29(5.20) | 24(7.59) | 16(3.70) | 151(6.68) | 23(0.88) | 43(16.48) |
| VAD | 6(4.51) | 6(1.18) | 1(0.89) | 10(1.79) | 9(2.85) | 8(1.85) | 14(0.62) | 14(0.54) | 14(5.36) |
| **Admission diagnosis, n(%)** |  |  |  |  |  |  |  |  |  |
| Sepsis | 28(21.05) | 154(30.20) | 44(39.29) | 174(31.18) | 82(25.95) | 157(36.34) | 1145(50.66) | 1431(54.72) | 28(10.73) |
| Respiratory | 5(3.76) | 82(16.08) | 26(23.21) | 113(20.25) | 56(17.72) | 90(20.83) | 575(25.44) | 707(27.04) | 23(8.81) |
| Cardiac arrest | 4(3.01) | 39(7.65) | 14(12.50) | 20(3.58) | 25(7.91) | 31(7.18) | 61(2.70) | 102(3.90) | 6(2.30) |
| GI Bleeding | 8(6.02) | 36(7.06) | 14(12.50) | 55(9.86) | 34(10.76) | 87(20.14) | 378(16.73) | 482(18.43) | 9(3.45) |
| Hypovolemia | 6(4.51) | 26(5.10) | 3(2.68) | 14(2.51) | 1(0.32) | 1(0.23) | 51(2.26) | 64(2.45) | 3(1.15) |
| Rhythm disturbance | 14(10.53) | 42(8.24) | 5(4.46) | 17(3.05) | 7(2.22) | 4(0.93) | 35(1.55) | 35(1.34) | 4(1.53) |
| Trauma | 3(2.26) | 7(1.37) | 3(2.68) | 6(1.08) | 3(0.95) | 11(2.55) | 39(1.73) | 35(1.34) | 3(1.15) |
| CHF | 14(10.53) | 32(6.27) | 4(3.57) | 60(10.75) | 13(4.11) | 10(2.31) | 40(1.77) | 59(2.26) | 31(11.88) |
| CABG | 10(7.52) | 14(2.75) | 2(1.79) | 25(4.48) | 59(18.67) | 39(9.03) | 141(6.24) | 42(1.61) | 55(21.07) |
| AMI | 8(6.02) | 34(6.67) | 2(1.79) | 36(6.45) | 8(2.53) | 11(2.55) | 39(1.73) | 51(1.95) | 18(6.90) |
| **Clinical outcomes** |  |  |  |  |  |  |  |  |  |
| ARF, n (%) | 51(38.35) | 166(32.55) | 52(46.43) | 266(47.67) | 102(32.28) | 151(34.95) | 728(32.21) | 963(36.83) | 115(44.06) |
| Ventilation LOS (days) | 5.13±4.36 | 5.73±5.75 | 4.67±4.42 | 5.80±5.18 | 5.98±6.40 | 6.69±7.29 | 6.09± 6.45 | 6.22±6.08 | 5.95± 6.34 |
| ICU mortality, n (%) | 20(15.04) | 86(16.86) | 45(40.18) | 145(25.99) | 145(45.89) | 259(59.95) | 405(17.92) | 664(25.39) | 61(23.37) |
| ICU LOS (hours) | 192.29±237.00 | 157.33±232.65 | 123.00±121.96 | 173.31±187.65 | 256.09±313.52 | 224.63±298.46 | 155.57±192.96 | 150.46±181.25 | 238.18±238.11 |
| Hospital mortality, n (%) | 31(23.31) | 112(21.96) | 46(41.07) | 191(34.23) | 154(48.73) | 282(65.28) | 541(23.94) | 845(32.31) | 74(28.35) |
| Hospital LOS (days) | 14.10±12.27 | 12.00±10.98 | 9.99±9.50 | 13.05±10.62 | 15.65±12.91 | 13.52±12.92 | 12.91± 10.67 | 12.10± 10.45 | 17.32±11.91 |

CVD: Cardiovascular diseases, BMI: Body Mass Index, APACH-IV: acute physiology and chronic health evaluation score IV, APS-II: simplified acute physiology score II, CHF: congestive heart failure, RMI: Remote Myocardial Infarction, CABG: Coronary Artery Bypass Grafting, PCI: Procedural Coronary Intervention, COPD: chronic obstructive pulmonary disease, MICU: medical intensive care unit, CCU: Coronary Care Unit, CTICU: cardiothoracic intensive care unit, CSICU: Cardiac Surgery Intensive Care Unit, PAC: Pulmonary artery catheter, IABP: intra-aortic balloon pump, VAD: Ventricular assist device, AMI: Acute Myocardial Infarction, ARF: acute renal failure, LOS: length of stay, GI: gastrointestinal.
